# Sjögren’s, Renal Tubular Acidosis and Osteomalacia - An Asian Indian Series

**DOI:** 10.2174/1874312901812010114

**Published:** 2018-07-24

**Authors:** Pulukool Sandhya, Debashish Danda, Simon Rajaratnam, Nihal Thomas

**Affiliations:** 1 Department of Clinical Immunology and Rheumatology, Christian Medical College and Hospital, Vellore-632004, India; 2 Department of Endocrinology, Diabetes and Metabolism, Christian Medical College and Hospital, Vellore-632004, India

**Sjögren’s, Renal Tubular Acidosis And Osteomalacia - An Asian Indian Series**

The Open Rheumatology Journal, **2014**, 8: 103-109

The correct keywords which are mentioned below:

Osteomalacia, Pseudofractures, Renal Tubular Acidosis, Sjögren’s syndrome, Vitamin D.

The original keywords provided were: 

Osteomalacia, Pseudofractures, Renal Tubular Acidosis, Sj gren s syndrome, Vitamin D.

